# College anti-smoking policies and student smoking behavior: a review of the literature

**DOI:** 10.1186/s12971-017-0117-z

**Published:** 2017-02-01

**Authors:** Brooke L. Bennett, Melodi Deiner, Pallav Pokhrel

**Affiliations:** Cancer Prevention & Control Program, University of Hawaii Cancer Center, University of Hawaii at Manoa, 701 Ilalo St, Honolulu, HI96822 USA

**Keywords:** Young adults, Cigarette smoking, College, Policies

## Abstract

**Background:**

Currently, most college campuses across the U.S. in some way address on-campus cigarette smoking, mainly through policies that restrict smoking on campus premises. However, it is not well understood whether college-level anti-smoking policies help reduce cigarette smoking among students. In addition, little is known about policies that may have an impact on student smoking behavior. This study attempted to address these issues through a literature review.

**Methods:**

A systematic literature review was performed. To identify relevant studies, the following online databases were searched using specific keywords: Ovid MEDLINE, PsycINFO, PubMed, and Google Scholar. Studies that met the exclusion and inclusion criteria were selected for review. Studies were not excluded based on the type of anti-smoking policy studied.

**Results:**

Total 11 studies were included in the review. The majority of the studies (54.5%) were cross-sectional in design, 18% were longitudinal, and the rest involved counting cigarette butts or smokers. Most studies represented more women than men and more Whites than individuals of other ethnic/racial groups. The majority (54.5%) of the studies evaluated 100% smoke-free or tobacco-free campus policies. Other types of policies studied included the use of partial smoking restriction and integration of preventive education and/or smoking cessation programs into college-level policies. As far as the role of campus smoking policies on reducing student smoking behavior is concerned, the results of the cross-sectional studies were mixed. However, the results of the two longitudinal studies reviewed were promising in that policies were found to significantly reduce smoking behavior and pro-smoking attitudes over time.

**Conclusion:**

More longitudinal studies are needed to better understand the role of college anti-smoking policies on student smoking behavior. Current data indicate that stricter, more comprehensive policies, and policies that incorporate prevention and cessation programming, produce better results in terms of reducing smoking behavior.

## Background

Tobacco use, especially cigarette smoking, continues to remain a leading preventable cause of mortality in the United States (U.S.). Across different age-groups, young adults (18–29 year olds) tend to show the highest prevalence of cigarette smoking [1]. For example, past-30-day prevalence of cigarette smoking among 18–24 year olds is 17%, whereas the prevalence is approximately 9% among high school students [2]. Although most smokers initiate cigarette smoking in adolescence, young adulthood is the period during which experimenters transition into regular use and develop nicotine dependence [1]. Young adulthood is also the period that facilitates continued intermittent or occasional smoking [3], neither of which is safe. In addition to the possibility that intermittent smokers may show escalation in nicotine dependence, intermittent smoking exposes individuals to carcinogens and induces adverse physiological consequences [4].

Research [5] shows that smokers who quit smoking before the age of 30 almost eliminate the risk of mortality due to smoking-induced causes. Thus smoking prevention and cessation efforts that target young adults are of importance. Traditionally, tobacco-related primary prevention efforts have mostly focused on adolescents [6] and have utilized mass media as well as school and community settings [7, 8]. This is only natural given that most smoking initiation occurs in adolescence. However, primary and secondary prevention efforts focusing on young adults have been less common. This is particularly of concern because tobacco industry is known to market tobacco products strategically to promote tobacco use among young adults by integrating tobacco use into activities and places that are relevant to young adults [9].

As more and more young adults attend college [10], college campuses provide a great setting for primary and secondary smoking prevention as well as smoking cessation efforts targeting young adults. According to the American College Health Association [11], approximately 29% U.S. college students report lifetime cigarette smoking and 12% report past-30-day smoking. Currently, most college campuses across the U.S. in some way address on-campus cigarette smoking, mainly through policies that restrict smoking [12, 13]. One of the main reasons why such policies are considered important is the concern about students’ exposure to secondhand tobacco smoke [14]. Therefore, at their most rudimentary forms, such policies tend to be extensions of local- or state-level policies restricting smoking in public places [15]. However, some colleges may take a more comprehensive approach, by integrating, for example, smoke-free policies with anti-smoking campaigns and college-sponsored cessation services [16]. Further, some colleges may implement plans to enhance enforcement of and compliance to the smoke-free policies [17–19].

At present, there are a number of questions related to college-level anti-smoking policies that need to be examined carefully in order to scientifically inform how colleges can be better utilized to promote smoking prevention and cessation among young adults. Besides the degree of variation in anti-smoking policies, there are questions about students’ compliance with such policies and whether such policies have influence on students’ attitudes and behavior related to cigarette smoking. Past reviews of the studies on the effects of tobacco control policies in general (e.g., not specific to college populations) [20–22] emphasize the need for a review such as the current study. Wilson et al. [20] found that interventions involving smoke-free public places, mostly restaurants/bars and workplaces, showed a moderate to low effect in terms of reducing smoking prevalence and promoting smoking cessation. The review included three longitudinal studies, none of which showed that the policies had an effect on smoking cessation. Fichtenberg & Glanz [21] focused on smoke-free workplaces and found that the effects of such policies seemed to depend on their strength. That is, 100% smoke-free policies were found to reduce cigarette consumption and smoking prevalence twice as much as partial smoke-free policies that allowed smoking in certain areas. In a recent exhaustive review, Frazer et al. [22] found that although national restrictions on smoking in public places may improve cardiovascular health outcomes and reduce smoking-related mortality, their effects on smoking behavior appear inconsistent. There are reasons why college anti-smoking policies may be more effective than policies focused on restaurant/bars or even workplaces. For example, students tend to spend the majority of their time on campus premises. In fact, in the case of 4-year colleges, a large number of students live on or around campus premises. Strong anti-smoking policies may deter students from smoking by making, for example, smoking very inconvenient. However, the current state of research on college anti-smoking policies and student smoking behavior is not well documented.

The purpose of the current study is to systematically review quantitative studies that have investigated the impact of college-level anti-smoking policies on students’ attitudes towards tobacco smoking and smoking behavior. In the process, we intend to highlight the types of research designs used across studies, the types of college and student participants represented across studies, and the studies’ major findings. A point to note is that this review’s focus is on anti-smoking policies and cigarette smoking. Although the review does assess tobacco-free policies in general, our assumption at the outset has been that most studies in the area have had a focus on smoke-free policies and smoking behavior because of the emphasis on secondhand smoke exposure. Smoke-free and tobacco-free policies are different in that smoke-free policies have traditionally targeted smoking only whereas tobacco-free policies that have targeted tobacco use of any kind, including smokeless tobacco [23]. Both types of policy could be easily extended to incorporate new tobacco products such as the electronic nicotine delivery devices, commonly known as e-cigarettes. Given that e-cigarettes are a relatively new phenomenon in the process of being regulated, we assumed that the studies eligible for the current review might not have addressed e-cigarette use, although if addressed by the studies reviewed, we were open to addressing e-cigarettes and e-cigarette use or vaping in the current review.

## Methods

### Study selection

We searched Ovid MEDLINE (1990 to June, 2016), PubMed (1990 to June, 2016), PsycINFO (1990 to 2013), and Google Scholar databases to identify U.S.-based peer-reviewed studies that examined the effects of college anti-smoking policies on young adults’ smoking behavior. Searches were conducted by crossing keywords “college” and “university” separately with “policy/policies” and “smoking”, “tobacco”, “school tobacco”, “smoke-free” “smoking ban,” and “tobacco free.” Article relevance was first determined by scanning the titles and abstracts of the articles generated from the initial search. Every quantitative study that dealt with college smoking policy was selected for the next round of appraisal, during which, the first and the last authors independently read the full texts of the articles to vet them for selection. Studies were selected for inclusion in the review if they met the following criteria: studies 1) were conducted in the U.S. college campuses, including 2- and 4-year colleges and universities; 2) were focused on young adults (18–25 year olds); 3) focused on implementation of college-level smoking policies; 4) were quantitative in methodology (e.g., case studies and studies based on focus groups and interviews were excluded); and 5) directly (e.g., self-report) or indirectly (e.g., counting cigarette butts on premises) assessed the cigarette smoking behavior. References and bibliographies of the articles that met the inclusion criteria were also carefully examined to locate additional, potentially eligible studies.

### Review

Selected studies were reviewed independently by the first and the last authors in terms of study objectives, study design (i.e., cross-sectional or longitudinal), data collection methods, participant characteristics, U.S. region where the study was conducted, college type (e.g., 2- year vs. 4-year), policies examined and the main study findings. The review results independently compiled by the two authors were compared and aggregated after differences were sorted out and a consensus was reached.

## Results

### Study characteristics

**Fig. 1:**
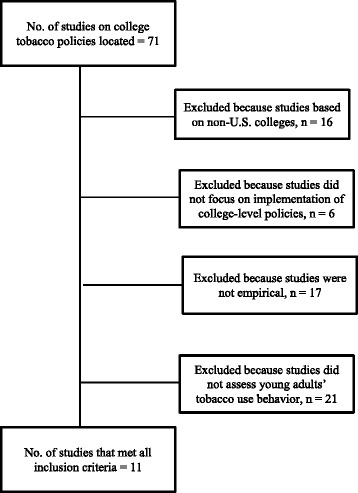
Chart depicting selection of the final set of articles reviewed

Figure 1 depicts the path to the final set of articles selected for review. Initial searches across databases resulted in total 71 titles and abstracts related to college smoking policies. Of these, 49 were deemed ineligible at the first phase of evaluation. The remaining 22 articles were evaluated further, of which, 11 were excluded eventually. Two studies [24, 25] were excluded because these studies did not assess students’ tobacco use behavior. One study [26] was excluded because it was not quantitative. Five studies [17–19, 27, 28] were excluded because the studies focused on compliance to existing smoking policies and did not assess the impact of policies on behavior. One study [15] was excluded because although it studied college students, the smoking policies examined were county-wide rather than college-level. Two studies [29, 30] were excluded because their samples consisted of college personnel rather than students. Thus, a total of 11 studies were included in the current review.

Table 1 summarizes the selected studies in terms of research purpose, study design, subjects, type of college, region, policies and findings. The majority of the studies were conducted in the Midwestern (*n* = 3; 27.3%) or Southeastern United States (*n* = 3; 27.3%). Other regions represented across studies were Southern (*n* = 2; 18.1%), Northwestern (*n* = 2; 18.1%), and Western United States (*n* = 1; 9.1%). Six studies (54.5%) included predominantly White participants (i.e., greater than 70%), and 2 studies (18%) included predominantly female participants. Nationally, women and Whites comprise 56% and 59% of the U.S. college student demographics, respectively [10]. Two studies (18.1%) assessed smoking behavior indirectly by counting cigarette butts on college premises, counting the number of individuals smoking cigarettes in campus smoking “hotspots,” or counting the number of smokers who utilized smoking cessation services. Across studies, the sample size ranged between *N* = 36 and *N* = 13,041. The mean and median sample sizes across studies were 3102 (SD = 4138) and 1309, respectively. Participants tended to range between 18 and 30 years in age. The majority of the studies (*n* = 6; 54.4%) were cross-sectional in design. Only 2 (18%) of the studies were longitudinal. The majority of the studies were conducted at 4-year colleges (*n* = 10; 90.9%). Only 1 study was conducted at a 2-year college (*n* = 1; 9.1%).

**Table 1:** Study characteristics and main study findings

Study	Purpose	Study Design	Methods	Policy type	Subjects	College type	Region	Findings
Borders et al. [31]	To determine the association between university tobacco control policies and students’ smoking behavior	Cross-sectional	Self-report	Prohibit on - campus sales and distribution of tobacco products Restrict smoking to 20 ft from building entrances Prohibit smoking in residence halls Clearly identify non-smoking areas Provide preventive education Provide smoking cessation classes	*N* = 13,041 M age NP, majority 18–22 years old; 61% F; 74% W, 11.7% H, 2.6 B, 11.4% O	4 year	South	Having preventive education program on campus was associated with lower odds of smoking. Presence of smoking cessation programs and designated smoking areas were associated with higher odds of smoking. Policies governing the sales and distribution of cigarettes were not associated with smoking.
Braverman et al. [33]	To determine the extent of outdoor tobacco smoke exposure and identify correlates of policy support a year after smoke-free policy was enactment	Cross-sectional	Self-report	Smoke-free campus	*N* = 3,994 M age NP, majority 18–25 years old; 45.5% F, 53.5% M, 77.5% W, 0.5% B, 9.2% A, 0.5% AI/AN, 0.4% NH/PI, 6.5% MI, 5.6% O	4 year	Northwest	Enactment of policy led to smoking activity shifting to the campus periphery. Limited exposure to smoke near building entrances since the policy was enacted, but the majority of staff (55%) and students (77%) reported increased exposure near campus boundaries
Butler et al. [40]	To determine the associations between community and campus smoke-free policies and attitudes and behaviors of undergraduate alcohol drinkers, including motivation to quit smoking	Cross-sectional	Self-report	Smoke-free campus and smoke-free bars close to campus	*N* = 337 M age = 20.3 (SD = 1.6); 68% F; 92% W, 8% O	4 year	South	26% of the sample were current smokers 9% of the smokers reported that the smoke-free policy increased their motivation to quit 3% reported that the policy reduced their motivation to quit 88% reported that the policy had no effect on their motivation to quit smoking 82% reported that the policy had no effect on the number of cigarettes smoked daily
Fallin et al. [34]	To validate the Tobacco-Free Compliance Assessment Tool designed to assess compliance with tobacco-free campus policy	Cross-sectional	Cigarette butts and smokers were counted in hot spots	Tobacco-free campus	NA	4 year	Southeast	More cigarette butts were counted in areas not covered by the tobacco-free policy in the health care campus No relationship found between location covered by the policy and the number of cigarette butts on the main campus
Fallin et al. [16]	To assess the association between tobacco-free college policies and students’ tobacco smoking behavior and attitudes	Cross-sectional	Self-report	Smoke-free indoors only (campus) Designated outdoor smoking areas (campus) Smoke-free campus Tobacco-free campus	*N* = 1309 M age NP, majority 18–24 years old; 61% F; 14% W, 4.5% B, 21% A, 39% H, 9% 0	4 year	West	Recent smoking was highest among students on campuses with designated outdoor smoking, compared to campuses with other policies (e.g., tobacco free, smoke-free) Stronger policies were associated with reduced self-reported exposure to secondhand smoke Students on tobacco-free campuses were less likely to report intentions to smoke in the next 6 months compared with students in colleges with less comprehensive policies
Hahn et al. [16]	To assess the outcomes and costs associated with implementing a tobacco-free policy using 3 T approach (Tell, Treat, and Train)	Cross-sectional	Counting smokers using cessation services Self-port	Tobacco-free campus with smoking cessation service	Demographics for treatment seeking individuals NP *N* = 36 for survey; M age NP; 61% F; Ethnicity NP	4 year	Southeast	335 smokers received treatment after policy took effect over 2-year period compared with 33 smokers in the year preceding policy enactment Average number of Nicotine Replacement Therapy coupons redeemed per month after policy took effect was 41 compared with 10 before policy enactment Survey results indicated higher confidence in remaining smoke-free while on campus among current smokers who received treatment
Harris et al. [32]	To test the effects of a tobacco-free campus policy enforcement package	Longitudinal	Observers recorded smokers’ compliance before and after intervention	Restrict smoking to 25 ft from building entrances	*N* = 709 M age = 22.0 (SD NP); 53% F, 82% W	4 year	Northwest	The intervention had a significant effect on compliance: 33% compliance at baseline increased to 74% during the intervention week and to 54% at follow-up
Lechner et al. [36]	To assess the effectiveness of a campus-wide anti-tobacco intervention	Longitudinal	Self-report	Tobacco-free campus with smoking cessation service	*N* = 4947 M age = 20.5 (SD = 1.8) at baseline; 52.5% F, 82.8% W, 4.1% B, 2.3% A, 6% AI, 2.3% O	4 year	Midwest	Intervention was not effective in reducing general smoking prevalence but significantly reduced proportions of high-frequency smokers and low-frequency smokers Intervention had significant effects on reduced exposure to secondhand tobacco smoke Intervention had significant effects on reducing pro-smoking attitudes such as positive weight-loss expectancy
Lee et al. [35]	To examine differences in cigarette smoked on campus premises by campus policy strength	Cross-sectional	Cigarette butts were counted	Tobacco-free campus Designated smoking area	NA	2 year	Southeast	100% tobacco-free college campuses had significantly fewer cigarette butts on premises than campuses with no outdoor restrictions Butts on campuses with partial policies were not significantly different from campuses with 100% tobacco-free policies
Lochbihler et al. [41]	To determine social rewards associated with using designated smoking areas on college campuses	Cross-sectional	Self-report	Designated smoking area	Sample 1: *N* = 188 M age = 29 (SD = 8.4); 62.7% F; Ethnicity NP Sample 2: *N* = 94 M age = 29 (SD = 9.6); 60.6% F; Ethnicity NP	4 year	Midwestern	Social interaction while smoking on campus (as compared with smoking alone) significantly increased the perceived reward of smoking, looking forward to spending time in the campus smoking areas, and how many times the campus smoking areas were visited Although designated smoking areas may protect nonsmoking students from the dangers of secondhand smoke, these areas may increase the rewards associated with nicotine for the smokers who use them
Seo, Macy, Torabi, and Middlestadt [37]	To assess change in students’ attitudes and behaviors due to policy implementation	Longitudinal	Self-report	Smoke-free campus	*N* = 3266 M age = 20.0 (SD NP); 58% F; 86% W, 4% B, 6% A, 0.4% NH/PI, 0.2% AI/AN, 2.2% M	4 year	Midwest	Compared with the control condition, students exposed to smoke-free campus policy showed significant reduction in smoking behavior.

*M* Mean, *SD* Standard Deviation, *W* White, *B* Black/African American, *H* Hispanic, *A* Asian, *AI/AN* American Indian/Alaskan Native, *NH/PI* Native Hawaiian/Pacific Islander, *M* Mixed ethnicity, *O* Other ethnicity, *F* Female, *T* Transgender, *NA* Not applicable, *NP* Not Provided

Three studies (27%) focused on tobacco-free policies and 3 studies (27%) on smoke-free policies. Three studies (*n* = 3; 27.3%) compared the associations of differing policies on smoking behavior. One study [31] examined the relative impacts of policies utilizing preventive education, smoking cessation programs, and designated smoking areas or partial smoking restriction. Another study [32] implemented an intervention to increase adherence to a partial smoking policy (i.e., smoking ban within 25 ft of buildings). The intervention involved increasing anti-tobacco signage, moving receptacles, marking the ground, and distributing reinforcements and reminder cards.

### Anti-smoking policies and students’ smoking behavior

Table 1 lists the types of anti-smoking policies examined across studies and the corresponding findings. Major findings are as follows:

#### Partial smoking restriction

Borders et al. [31] compared colleges that utilized partial smoking restriction by providing “designated smoking areas” to curb smoking with college-level policies that incorporated preventive education and with those that provided smoking cessation courses only. Results indicated that the presence of preventive education was associated with lower odds of past-30-day smoking whereas the presence of designated smoking areas only or smoking cessation programs only was associated with higher odds of past-30-day smoking. Fallin et al. [16] found that college campuses with designated smoking areas tended to show higher prevalence of smoking, compared with campuses that enforced smoke-free and tobacco-free policies. Braverman et al.’s [33] findings indicate that enforcing smoke-free policies tends to reduce secondhand exposure close to college buildings but may increase smoking behavior on the campus periphery.

#### Smoke- and tobacco-free campuses

Fallin et al. [16] found that compared with policies that relied on partial smoking restriction, tobacco-free policies were associated with reduced self-reported exposure to secondhand smoke as well as students’ lower self-reported intentions to smoke cigarettes in the future. Studies [34, 35] consistently observed fewer cigarette butts or smokers in campuses under smoke-free policies compared with campuses without smoke-free policies. Prevalence of cigarette butts was likely to be inversely related to policy strength [35]. A study that monitored smokers’ behavioral compliance to smoke-free policies [32] indicated that interventions to promote compliance, such as use of signage, are likely to be effective in improving compliance and reducing student smoking in areas were the policy is enforced.

Lechner et al. [36] conducted assessments at a single college campus before and after a tobacco-free policy went into implementation. The policy, which also involved making smoking cessation services available campus-wide, was found to reduce proportions of high- and low-frequency smokers, pro-smoking attitudes (i.e., weight loss expectancy), and exposure to second-hand tobacco smoke [36]. The study did not find an effect on smoking prevalence. Seo et al. [37] followed a similar design where a policy intervention was evaluated based on pretest and posttest surveys. However, this study [37] included a “control” campus where similar assessments as in the “treatment” campus were conducted but no intervention was implemented. The study found that compared with the control campus, the campus that implemented smoke-free policies showed an overall decrease in smoking prevalence.

#### Other policies

Borders et al. [31] did not find policies governing the sales and distribution of cigarettes on campus to be associated with smoking behavior. Hahn et al. [38] found that college smoking policies that integrate smoking cessation services may increase the use of such services as well as promote smoking cessation. This study kept track of students who utilized the smoking cessation service offered by a college after the policy offering such a service was enacted. Sixteen months after the policy was first implemented, smokers who utilized the service were surveyed. Based the results it was estimated that approximately 9% of them had quit smoking.

## Discussion

To our knowledge, this is the first study to systematically review studies examining the effects of anti-smoking policies on smoking behaviors among U.S. college students. We found that such studies are severely limited. Only 11 studies met the inclusion criteria in the present review, although the review appeared to encompass all policies aimed at smoking behavior on college campuses. Thus, this review stresses the need for increased smoking policy and smoking behavior research on college campuses.

Rigorous evaluation of existing college anti-tobacco policies are needed to refine and improve the policies so that national-level efforts to reduce tobacco use among young adults are realized. Key initiatives at the national level have recognized the importance of mobilizing college campuses in the fight against tobacco use. For example, in September 2012 several national leaders involved in tobacco control efforts, in collaboration with the ACHA, came together to launch the Tobacco-Free College Campus Initiative (TFCCI) [39]. The TFCCI aims to promote and support the use of college-level anti-tobacco policies as a means to change pro-tobacco social norms on campuses, discourage tobacco use, protect non-smokers from second-hand exposure to tobacco smoke and promote smoking cessation. The ACHA’s position statement [11] regarding college tobacco control recommends a no tobacco use policy aimed towards achieving a 100% indoor and outdoor campus-wide tobacco-free environment.

We found that the majority of studies on smoking policies were cross-sectional in nature. Researchers relied upon students to report their smoking behavior or their observations of other students’ smoking behavior after a smoke-free or tobacco-free policy had been implemented. It is difficult to draw conclusions about an anti-smoking policy’s ability to change smoking behavior without knowing the smoking behavior prior to policy implementation. This domain of research would benefit from additional longitudinal studies. Ideally, research studies should collect data before the policy is implemented, immediately after, and at follow-up time points.

We found inconsistencies in the measurement of smoking behavior across studies. Two studies [34, 35] counted cigarette butts, one study [38] counted people seeking tobacco dependence treatment, one study [32] counted smokers violating policy, and seven studies [16, 31, 36, 37, 40, 41] relied upon self-report of smoking behavior. Another study [33] used survey methods to obtain participants’ response on other students’ smoking behavior. Counting cigarette butts has been validated as an effective measure of smoking behavior [19], especially when validating compliance to an anti-smoking policy, and self-report measures are commonly used in public health research [42]. Despite the validity and feasibility of these measures, the lack of a consistent measurement tool makes comparing effectiveness of anti-smoking policies on smoking behaviors across campuses difficult. Research in this domain would benefit from a consistently used measurement of smoking behaviors.

Although the reviewed studies represented diverse U.S. regions, the majority of the research was set in the Southeastern and Midwestern United States; Northeastern and Southwestern regions were not represented. Only one of the reviewed studies reported a sample that contained less than 50% White participants. Across studies, the minority group most represented was Asian American; but only one of the reviewed studies [16] included 20% or more Asian Americans. Relatively few studies included or reported Hispanic participants, although Hispanics are the largest minority group in the United States [43]. None of the reviewed studies included 20% or more Black participants. Only three studies [33, 36, 37] included American Indian/Alaska Natives and in only one of those studies [32] was the proportion greater than one percent. Only two studies [33, 37] included Pacific Islanders, and in both the proportion was less than one percent. Clearly, more research is needed on minority populations, specifically Black, Hispanic, Native Hawaiian/Pacific Islander, American Indian/Alaska Native students and the subgroups commonly subsumed under these ethnic/racial categories. The U.S. college student demography is ethnically/racially diverse [10], comprising 59% Whites. The remaining 44% include various minority groups. Thus, for research on U.S. college students across the nation, studies with more ethnically/racially diverse student samples are needed.

The review findings were helpful in elucidating the types of tobacco policies being implemented on college campuses and their effects on the smoking behavior of U.S. college students. Mainly, three types of smoking policies were studied: smoke-free policies, tobacco-free policies and policies that enforced partial smoking restriction, including prohibition of smoking within 20–25 ft of all buildings and providing designated smoking areas. Indeed, campus-wide indoor and outdoor tobacco-free policy is considered a gold-standard for college campus tobacco control policy [11]. But only one study [16] compared tobacco-free and smoke-free policies. Other policies such as governing the sale and distribution of tobacco products, preventive education programs, and smoking cessations programs were also studied, but to a lesser extent. In general, interventions regarding the implementation of smoking policies on college campuses were difficult to find in the existing literature.

The combined results of the studies reviewed suggest that stricter smoking policies are more successful in reducing the smoking behavior of students. Tobacco-free and smoke-free policies were linked with reduced smoking frequency [16, 36, 37], reduced exposure to second-hand smoke [16, 36], and a reduction in pro-smoking attitudes [36]. Implementation of a campus-wide tobacco-free or smoke-free policy combined with access to smoking cessation services was also associated with increased quit attempts [38, 40] and treatment seeking behaviors [38]. It appears that 100% smoke-free policies are not only successful in reducing smoking rates, but also have strong support from students and staff members alike [33]. These results remained consistent when compared to less comprehensive tobacco control policies, which was evidenced by student report and the number of cigarette butts found on campus [34, 35].

There was one important consistent exception to the general success of anti-smoking policies: designated smoking areas. All three studies which included designated smoking areas [16, 31, 41] found that designated smoking areas were associated with higher rates of smoking compared with smoke-free or tobacco-free policies. Designated smoking areas were also associated with the highest rates of recent smoking [16]. Lochbihler, Miller, and Etcheverry [41] proposed that students using the designated areas were more likely to experience positive effects of social interaction while smoking. They found that social interaction while smoking on campus significantly increased the perceived rewards associated with smoking and the frequency of visits to designated smoking areas [41].

None of the studies included in this review addressed new and emerging tobacco products such as e-cigarettes. This is understandable given that the surge in e-cigarette use is relatively new and in general there have only been a few studies examining the effects of anti-smoking policies on student smoking behavior, which has been the focus of this review. However, going forward, it will be crucial for studies to examine how campus policies are going to handle e-cigarette use, including the enforcement of on-campus anti-smoking policies given the new challenges posed by e-cigarette use [44]. For example, e-cigarette use is highly visible, the smell of the e-cigarette vapor does not linger in the air for long and e-cigarette consumption does not result in something similar to cigarette butts. These characteristics are likely to make the monitoring of policy compliance more difficult. Moreover, because of the general perception among e-cigarette users that e-cigarette use is safer than cigarette smoking, compared with cigarette smokers smoking cigarettes, e-cigarette users might be more likely to use e-cigarettes in public places. The fact that the TFCCI strongly recommends the inclusion of e-cigarettes in college tobacco-free policies [39] bodes well for the future of college health.

The current study has certain limitations. It is possible that this review might have missed a very small number of eligible studies. We believe that the literature searches we completed were thorough. However, new studies are regularly being published and the possibility that a new, eligible study may have been published after we completed our searches cannot be ignored. In addition, we may not have tapped eligible studies that were in press during our searches. If indeed a few eligible studies were not included in our review, the non-inclusion may have biased our results somewhat, although it is difficult for us to speculate the nature of such a bias. Hence, we recommend that similar studies need to be conducted in the future to periodically review the literature. Second, non-peer-reviewed articles or book chapters were excluded from this review. Despite the potential relevance of non-peer-reviewed materials, the choice was made to limit the inclusion in order to maintain scientific rigor of the review. However, it is possible that some data pertinent to the review might have been overlooked because of this, thus increasing the possibility of introducing a bias to the current findings. Third, this study focused on anti-smoking policies. Although we used “tobacco free” as search terms, “smoking” dominated our search strategies. Thus our results are more pertinent to cigarette smoking than other tobacco products and may not generalize to the latter. Lastly, in order to be as inclusive as possible, we reviewed three studies [32, 35, 38] that focused on more on compliance to anti-smoking policy than on the effect of policy on student smoking behavior. The findings of these studies may not be comprehensive in regard to student smoking behavior, even though they are indicative of the success of the policies under examination.

## Conclusions

Despite limitations, this study is significant for increasing the understanding of smoking policies on U.S. college campuses and their effects on the smoking behavior of college students. We found that research on smoking policies on U.S. college campuses is very limited and is an area in need of additional research contribution. Within existing research, the majority used samples that were primarily White females. More diverse samples are needed. Future research should also report the full racial/ethnic characteristics of their samples in order to identify where representation may be lacking. Future research would benefit from longitudinal and interventional studies of the implementation of smoking policies. The majority of current research is cross-sectional, which does not provide the needed data in order to make causal statements about anti-smoking policies. Lastly, existing research was primarily conducted at 4-year colleges or universities. Future research would benefit from broadening the target campuses to include community colleges and trade schools. Community colleges provide a rich and unique opportunity to collect data on a population that is often older and more racial diverse than a typical 4-year college sample [45]. Also, there is at present a need to understand through research how evidence-based implementation and compliance strategies can be utilized to ensure policy success. A strong policy on paper does not often translate into a strong policy in action. Thus, comparing policies on the strength of written documents alone is not enough; policies need to be compared on the extent to which they are enforced as well as the impact they have on student behavior.

This review may be of particular interest to college or universities in the process of making their own anti-smoking policies. The combined results of the existing studies on the impact of anti-smoking policies on smoking behaviors among U.S. college students can help colleges and universities make informed decisions. The existing research suggests that stricter policies produce better results for smoking behavior reduction and with smoking continuing to remain a leading preventable cause of mortality in the U.S. across age-groups [1], college and university policy makers should take note. Young adults (18–25 year olds) show the highest prevalence of cigarette smoking [1], which places colleges and universities in the unique position to potentially intervene through restrictive anti-smoking policies on campus.
